# Effectiveness of Panretinal Photocoagulation Plus Intravitreal Anti-VEGF Treatment Against PRP Alone for Diabetic Retinopathy: A Systematic Review With Meta-Analysis

**DOI:** 10.3389/fendo.2022.807687

**Published:** 2022-03-29

**Authors:** Wuyue Zhang, Jinsong Geng, Aimin Sang

**Affiliations:** ^1^ School of Medicine, Nantong University, Nantong, China; ^2^ Department of Medical Informatics, School of Medicine, Nantong University, Nantong, China; ^3^ Affiliated Hospital of Nantong University, Nantong, China

**Keywords:** panretinal photocoagulation, anti-vascular endothelial growth factor, diabetic retinopathy, meta-analysis, combination therapy

## Abstract

**Objective:**

To compare the efficacy and safety of panretinal photocoagulation (PRP) combined with intravitreal anti-vascular endothelial growth factor (anti-VEGF) against PRP monotherapy for diabetic retinopathy (DR).

**Methods:**

We searched Pubmed, Cochrane Library, Web of Science, Embase, and Science Direct Register of Controlled Trials from April 2011 to January 2021 to identify the randomized trials that compared the efficacy and safety between PRP combined with intravitreal anti-VEGF and PRP monotherapy for DR. We searched in the following databases between April 2011 and January 2021: Pubmed, Cochrane Library, Web of Science, Embase, and Science Direct without any restriction of countries or article type. The outcome measures were the best-corrected visual acuity (BCVA), neovascularization on the disc (NVD), neovascularization elsewhere (NVE), central macula thickness (CMT), and total retinal volume over time (FAS), and we also observed the adverse events (AEs) between the two groups.

**Results:**

A total of 351 studies were identified, of which 11 studies were included in this meta-analysis (N = 1,182 eyes). Compared with PRP monotherapy, PRP plus anti-VEGF combination treatment produced a mean reduction in BCVA in units of logMAR of -0.23 [95% CI -0.32, -0.15] or a mean improvement in BCVA in units of letters of 4.99 [95% CI 3.79, 6.19], and also yielded a mean reduction in NVD of -28.41 [95% CI -30.30, -26.52], in NVE of -1.33 [95% CI -1.52, -1.14], in CMT of -1.33 [95% CI -1.52, -1.14], or in total FAS. No significant difference was observed on the risk of AEs as vitreous hemorrhage, elevation in intraocular pressure, and cataract between the two different treatments.

**Conclusion:**

PRP with anti-VEGF combination treatment can achieve the ideal efficacy on DR by improving BCVA and NV regression, with no potential increased incidence of AEs, which proves that the combination therapy is an efficient therapeutic strategy that could improve the management of patients with DR.

## Introduction

Diabetic retinopathy (DR) has become a leading cause of visual impairment in working age in industrialized countries. DR can be divided into two classifications including non-proliferative diabetic retinopathy (NPDR) and proliferative diabetic retinopathy (PDR), of which PDR is the more advanced stage leading to visual impairment and loss in the nearly total absence of symptoms (1). In recent decades, there have been a variety of treatments for DR, among which panretinal photocoagulation (PRP) and anti-vascular endothelial growth factor (anti-VEGF) therapy are commonly used. VEGF is a kind of homodimeric glycoprotein, including PLGF, VEGF-A, VEGF-B, VEGF-C, and VEGF-D. VEGF can promote endothelial cell-specific mitogen, increase vascular permeability, and promote inflammation (2). Under the condition of high glucose, oxidative stress, hemodynamic changes, and inflammatory reactions are involved in the regulation of VEGF expression and increase the concentration of VEGF. VEGF increases capillary permeability by the phosphorylation of tight-junction protein, leading to macular edema and angiogenesis (3). Therefore, inhibition of VEGF has become an important part of anti-neovascularization therapy. Anti-VEGF agents available include bevacizumab, ranibizumab, and aflibercept and are gaining popularity by intravitreal injection while the mainstay of surgical treatment for PDR is Scatter, or panretinal, photocoagulation (4).

Both treatments have a positive effect on delaying the progression of DR, and they also have been combined many times in clinical practice. However, the efficacy of monotherapy and combined treatment may be different. Some adverse events can be found in the follow-up of both therapies. Administration or surgery methods may cause some ocular disorders such as vitreous hemorrhage, elevation in intraocular pressure, and cataract, and pesticide effects may result in some non-ocular disorders such as hypertension.

In order to inform in clinical decision-making which treatment can be preferred to use clinically, this meta-analysis examined 11 randomized controlled trials (RCTs), with patients from Canada, the United States, Pakistan, Brazil, and other countries, trying to compare the efficacy and safety of PRP with intravitreal anti-VEGF-combined treatment against PRP monotherapy.

## Methods

### Search Strategy and Selection Criteria

This meta-analysis is reported in accordance with the Systematic Reviews and Meta-Analyses (PRISMA) Statement. The registration number in PROSPERO is CRD42021254750.

We searched Pubmed, Cochrane Library, Web of Science, Embase, and Science Direct Register of Controlled Trials between April 2011 and January 2021 to identify the randomized trials that compared the efficacy and safety between PRP combined with intravitreal anti-VEGF and PRP monotherapy for DR. We selected several studies published between April 2011 and January 2021. We searched the following databases: Pubmed, Cochrane Library, Web of Science, Embase, and Science Direct without any restriction of countries or article type. The reference list of all selected articles is independently screened to identify additional studies left out in the initial search. We used the combined search strategy with text and MESH terms as follows: ((“Diabetic Retinopathy”[Mesh]) OR ((proliferative diabetic retinopathy[Title/Abstract]) OR PDR[Title/Abstract])) AND (((“retinal laser photocoagulation”[Mesh]) OR retinal laser photocoagulation[Title/Abstract])) AND (((((((VEGF[Title/Abstract]) OR anti VEGF [Title/Abstract]) OR anti-VEGF [Title/Abstract]) OR lucentis[Title/Abstract]) OR bevacizumab[Title/Abstract]) OR ranibizumab [Title/Abstract]) OR aflibercept[Title/Abstract])) AND (“Randomized Controlled Trial” [Publication Type]).

### Eligibility Criteria

Eligible studies are regarded as RCTs, comparing PRP plus anti-VEGF with PRP alone, reporting changes in best-corrected visual acuity (BCVA), neovascularization on the disc (NVD), neovascularization elsewhere (NVE), central macula thickness (CMT), or total retinal volume over time (FAS). We exclude studies in adherence to the following criteria: non-RCT studies, studies done in patients treated with drug monotherapy, and combination therapy on DR. We did not impose restriction on history of systemic treatment.

The primary outcomes were assessed as follows: the changes of BCVA (logMAR, letters), the regression of neovascularization defined as NVD (percentage of disc surface, DD%), NVE (disc diameter, DD), CMT (μm), and total retinal volume. The incidences of adverse events (AEs) were estimated as secondary outcomes, including vitreous hemorrhage, elevation in intraocular pressure, and cataract.

### Data Extraction

Two investigators collaborated on assessing these studies together, reviewing the titles and abstracts and retrieving studies satisfied for full-text assessment. Studies qualified were selected by two investigators with an agreement value of 99.4%. Disagreements were settled by a third investigator.

We extracted data in a standardized form from each selected study. The data included the first author and year of each study, total number of patients participated, age, follow-up period, details of therapeutic method, change in BCVA (mean [SD]), change in CMT (mean [SD]), change in BCVA (mean [SD]), change in NVD (mean [SD]), change in NVE (mean [SD]), change in total retinal volume (mean [SD]), and incidence of AEs.

In some cases where the data were ambiguous, we emailed some of the relevant corresponding authors to refine the data obtained.

### Statistical Analysis

We assessed the efficacy of PRP plus anti-VEGF agents versus PRP monotherapy on four outcomes: BCVA, regression of neovascularization (NVD, NVE, CMT), total retinal volume, and incidence of any AEs.

We analyzed BCVA, NVD, NVE, CMT, and total retinal volume as continuous variables and calculated pooled estimates of the mean differences (MD) in changes of BCVA, NVD, NVE, CMT, and total retinal volume of study groups and control groups by using a random-effect (RE) model to depict the characteristics of each group.

The incidence of AEs common in eligible studies was analyzed, from which an overall relative risk (ORR) was calculated. The pooled estimates of the relative risk with an RE model were also calculated.

As we divided studies into groups with data in common and the data set that counted changes in CMT contained a relatively large number of studies, which was seven, we assessed publication bias of the CMT group using Egger test and defined significant publication bias as a p value <0.05 (5).

### Risk-of-Bias Assessment

Cochran Q test and the I^2^ statistics were employed to assess heterogeneity between studies. I^2^ values greater than 50% represent moderate to high heterogeneity, while I^2^ values lower than 50% represent low heterogeneity (6). We investigated sources of heterogeneity through subgroup analyses. Review Manager (version 5.4, Cochrane Collaboration) and STATA (version 15.1, STATA Corp) were used for all statistical analyses. Further sensitivity analysis was also performed to confirm whether the heterogeneity had an impact on the conclusions.

## Results

**Figure 1:**
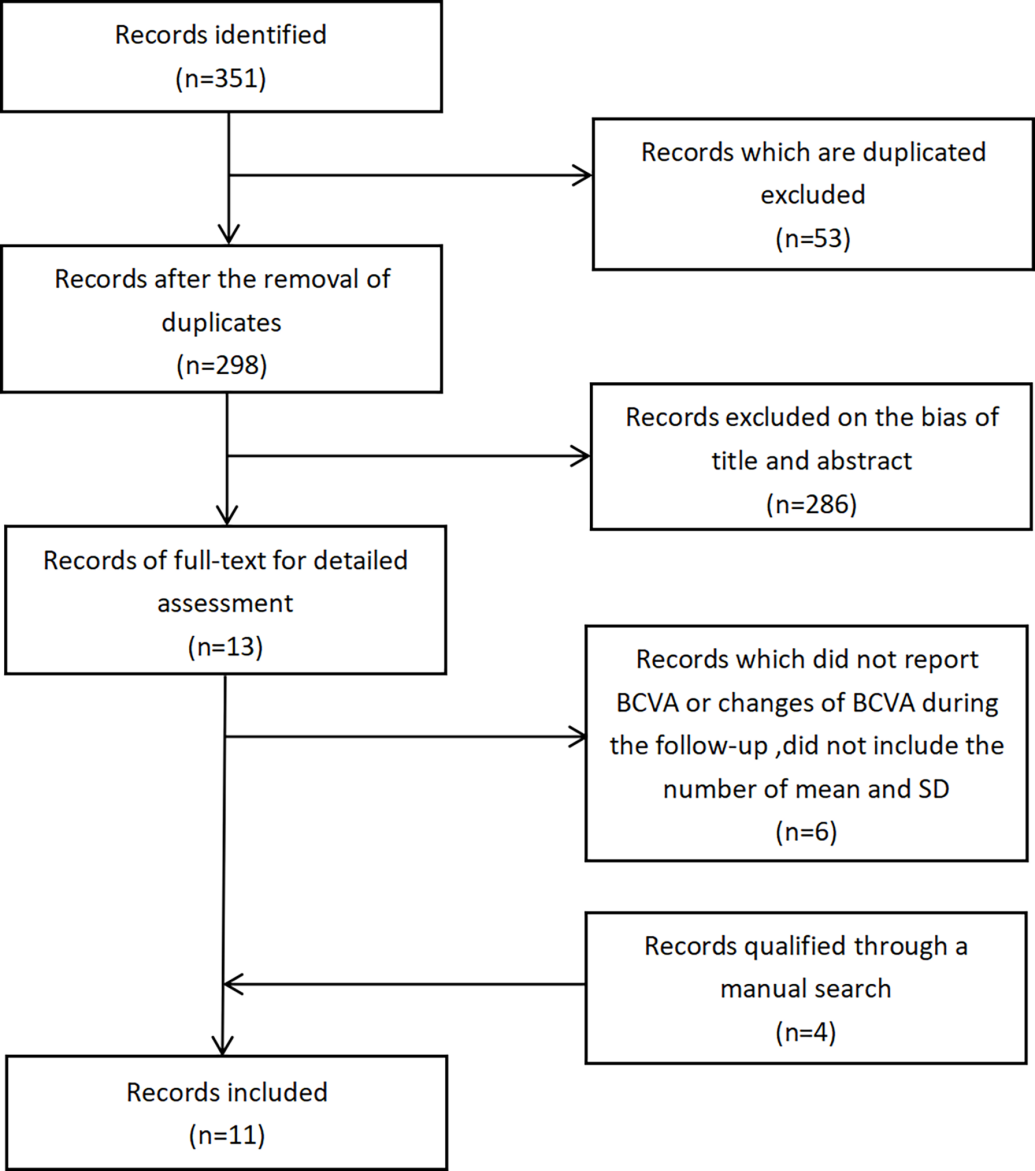
Process of study selection.

**Figure 2:**
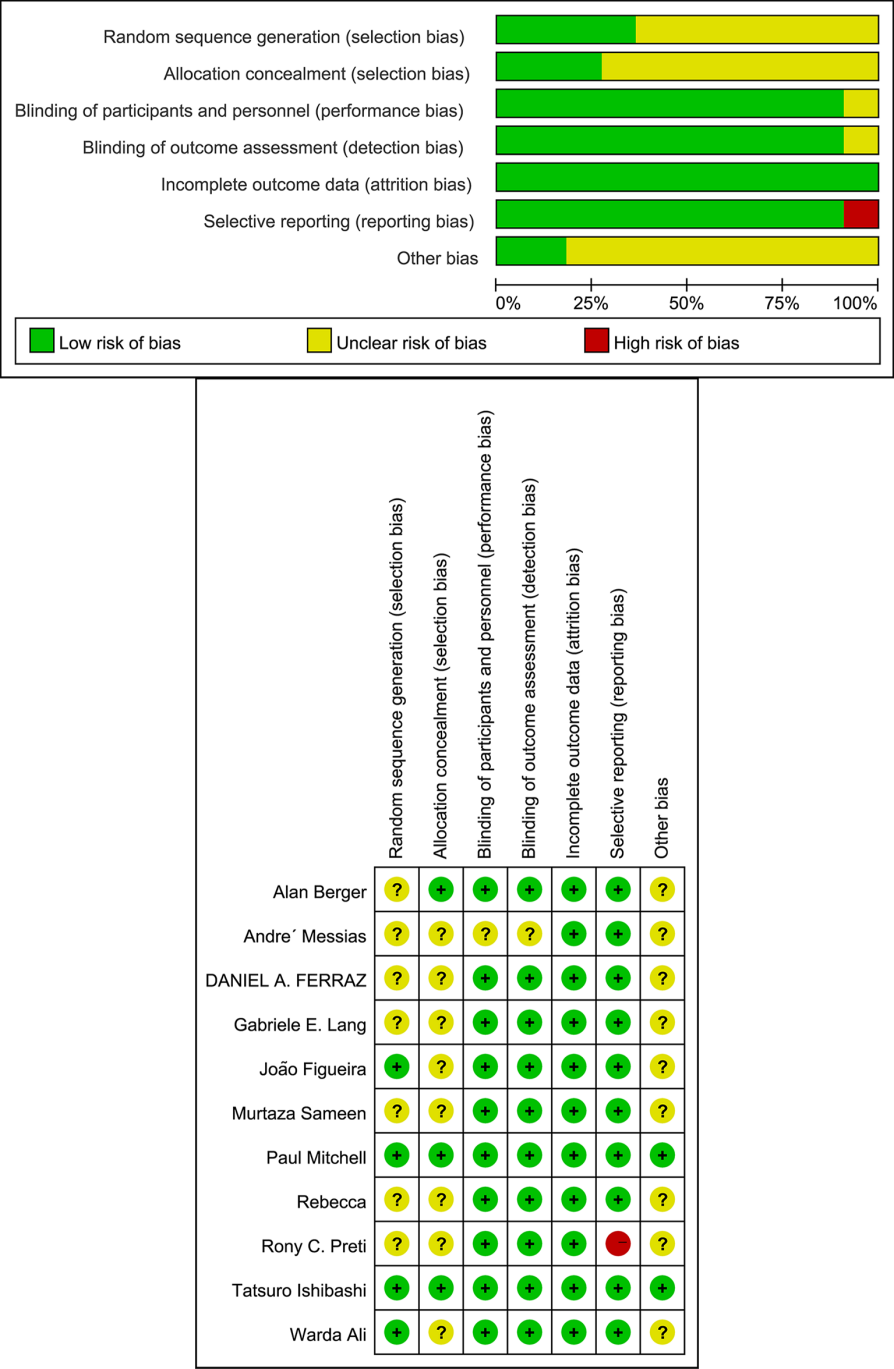
Risk of bias.

We identified 351 studies initially, of which 11 studies were included in this meta-analysis (**Figure 1**). The 11 studies were published from April 2011 to January 2021 (**Table 1**) (7–17). Follow-up duration ranges from 1 to 12 months. 9 studies (7, 8, 11–17) reported changes of BCVA, among which 4 studies (7, 12, 16, 17) specified the changes of BCVA in logMAR and 5 studies (8, 11, 13–15) in letters. 7 studies (9–11, 13, 15–17) measured the changes of CMT. 2 studies (7, 16) reported changes of NVD and NVE, which were specified in DD% and DD. One study (10) measured the change of mean total retinal volume over time in units of mm³ while the other study measured the changes of neovascularization total (NVT) in units of disc area (DA) (8). Adverse events were assessed by 6 studies (8, 10, 11, 13–15), of which 3 reported vitreous hemorrhage (8, 11, 15), 3 reported elevation in intraocular pressure (8, 10, 11), and 4 reported cataract (8, 11, 13, 14). One study (9) observed no adverse effects. We also assessed the risk of bias of all the 11 RCTs (**Figure 2**).

**Table 1:** Characteristics of included studies.

Study ID (first author)	Year	Design	Sample size (eyes)	Case/control (PRP plus IVB/PRP)	Average age/age range (case/control)	Details of anti-VEGF agent injection	Details of laser photocoagulation	Intraoperative and postoperative evaluating parameters	Follow-up periods (months)
Ali et al. (7)	2018	RCT	60	30/30	(40–52)/(53–65); (52.27 ± 6.8)	IVB (1.25 mg 0.05 ml) 15 days prior to PRP session	1,500–2,000 shots (200–500 μm spots), 0.05–0.1-s duration, 0.1 interval, 300–500-W power per episode, under topical anesthesia	BCVA, NVE, NVD	3
Figueira et al. (8)	2018	RCT	87	41/46	(58.8 ± 13.3)/(52.0 ± 11.9)	IVR, in month 0, month 1, and month 2 combined with the standard PRP treatment, that is, with 1, 2, or 3 laser sessions	1,200–1,600 scatter laser burns (500 μm spots)	BCVA, NVE, NVD, NVT, CMT, AEs	12
Preti et al. (9)	2017	RCT	38	19/19	(53.4 ± 9.3)/(53.4 ± 9.3)	IVB (1.25 mg/0.05 ml) 7 days prior to PRP session and at the end of the third PRP episode.	A spot size of 250 μm, an exposure time between 0.1 and 0.2-m, and a moderate intensity (200 to 500 mW)	CMT, choroidal thickness, AEs	1
Lang et al. (10)	2018	RCT	128	85/43	(63.5 ± 9.3)/(63.5 ± 10.5)	IVR (0.5 mg), 4 monthly	Focal laser and ranibizumab injections were administered on the same day with a minimum interval of 30 min between the two treatments	BCVA, FCS, and FCP thickness, CMT, AEs	6.2 ± 2.7
Berger et al. (11)	2015	RCT	145	73/72	(60.8 ± 10.2)/(62.8 ± 9.4)	IVR (0.5 mg ranibizumab intravitreal injections), 3 monthly injections followed by as-needed therapy	On day one. If required, the initial photocoagulation could be split into 2 sessions, 4 weeks apart	BCVA, CMT, VFQ-25 (Visual Function Questionnaire-25), AEs	12
Messias et al. (12)	2012	RCT	20	11/9	(59 ± 12)/(64 ± 8)	IVR ((0.5 mg/0.05 ml), at weeks 16 and 32	Six to eight hundred 500 lm spots were applied per session	BCVA, duration of diabetes, FLA	6
Mitchell et al. (13)	2011	RCT	229	118/111	(64.0 ± 8.15)/(63.5 ± 8.81)	IVR (0.5 mg), at months 0–2; treatment initiation phase	2 sessions, 4 weeks apart	BCVA, CMT, AEs	12
Ishibashi et al. (14)	2015	RCT	263	132/131	(61.2 ± 10.52)/(61.5 ± 9.68)	IVR (0.5 mg), on day 1 and continued monthly until stable vision was achieved	On day 1 (if needed, the first laser treatment session could be split into 2 sessions 4 weeks apart)	BCVA, AEs	12
Ferraz et al. (15)	2015	RCT	60	30/30	52.3 ± 7.8	IVR (0.5 mg) and were given again at Month 1	Full-scatter PRP treatment, performed in three sessions according to the ETDRS guidelines	BCVA, CMT, AEs	6
Rebecca et al. (16)	2021	RCT	76	38/38	(51.1 ± 5.9)/(50.7 ± 6.9)	IVB (1.25 mg/0.05 ml). In Group B subjects, first intravitreal bevacizumab injection (1.25 mg/0.05 ml) was given followed by PRP after the 1st week, likewise 2 more PRP episodes were done, and a second IVB injection was given at the end of the 3rd PRP session.	Three sessions of PRP were done with a 1-week interval; two thousand burns were applied at the 1st session with a 200-μm spot size, duration of 20-ms pulse, and from 400 to 500 mW power to achieve grayish burns; and further PRP was done at 1 and 2 months with supplementary 800–1,000 burns	BCVA, NVE, NVD, CMT	6
Sameen et al. (17)	2017	RCT	76	38/38	(57.47 ± 6.08)/(55.69 ± 6.58)	IVB (2.5 mg/0.1 ml) 1 day after PRP session and repeated monthly for 3 months.	Two thousand burns were applied at the 1st session with a 20-ms pulse duration, 200-μm spot size, and power ranging from 350 to 600 mW, titrating the burn intensity until a mild gray reaction was achieved, repeating at 4 weeks and 8 weeks with an additional 800–1,000 burns	BCVA, CMT	3

**Figure 3:**
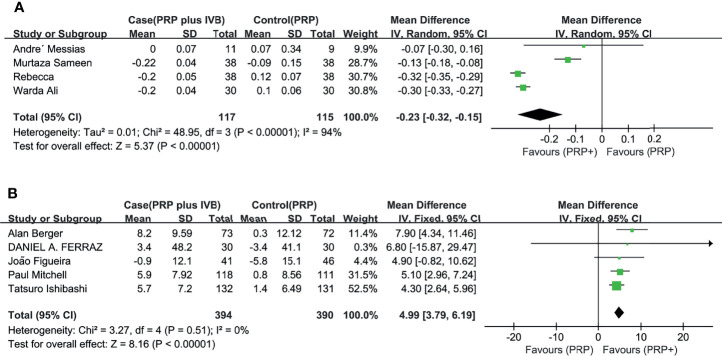
Meta-analyses of PRP+anti-VEGF treatment and PRP monotherapy, comparing changes of BCVA (logMAR, letters). **(A)** BCVA (logMAR), **(B)** BCVA (letters). Outcomes assessed: BCVA in studies that compared combination treatment with PRP monotherapy. The shaded area is the weight of the estimate in proportion to the overall effect.

**Figure 4:**
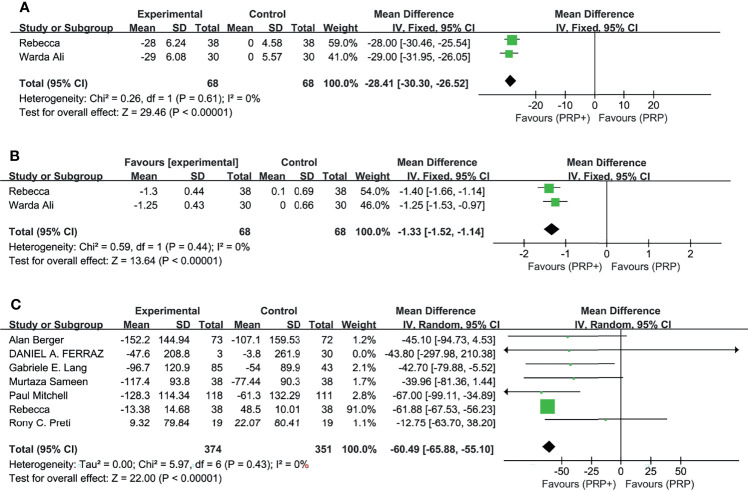
Meta-analyses of PRP+anti-VEGF treatment and PRP monotherapy, comparing changes of NVD (DD%), NVE (DD), and CMT (μm). **(A)** NVD (DD%), **(B)** NVE (DD), and **(C)** CMT (μm). Outcomes assessed: NVD, NVE, and CMT in studies that compared combination treatment with PRP monotherapy. The shaded area is the weight of the estimate in proportion to the overall effect.

**Figure 5:**
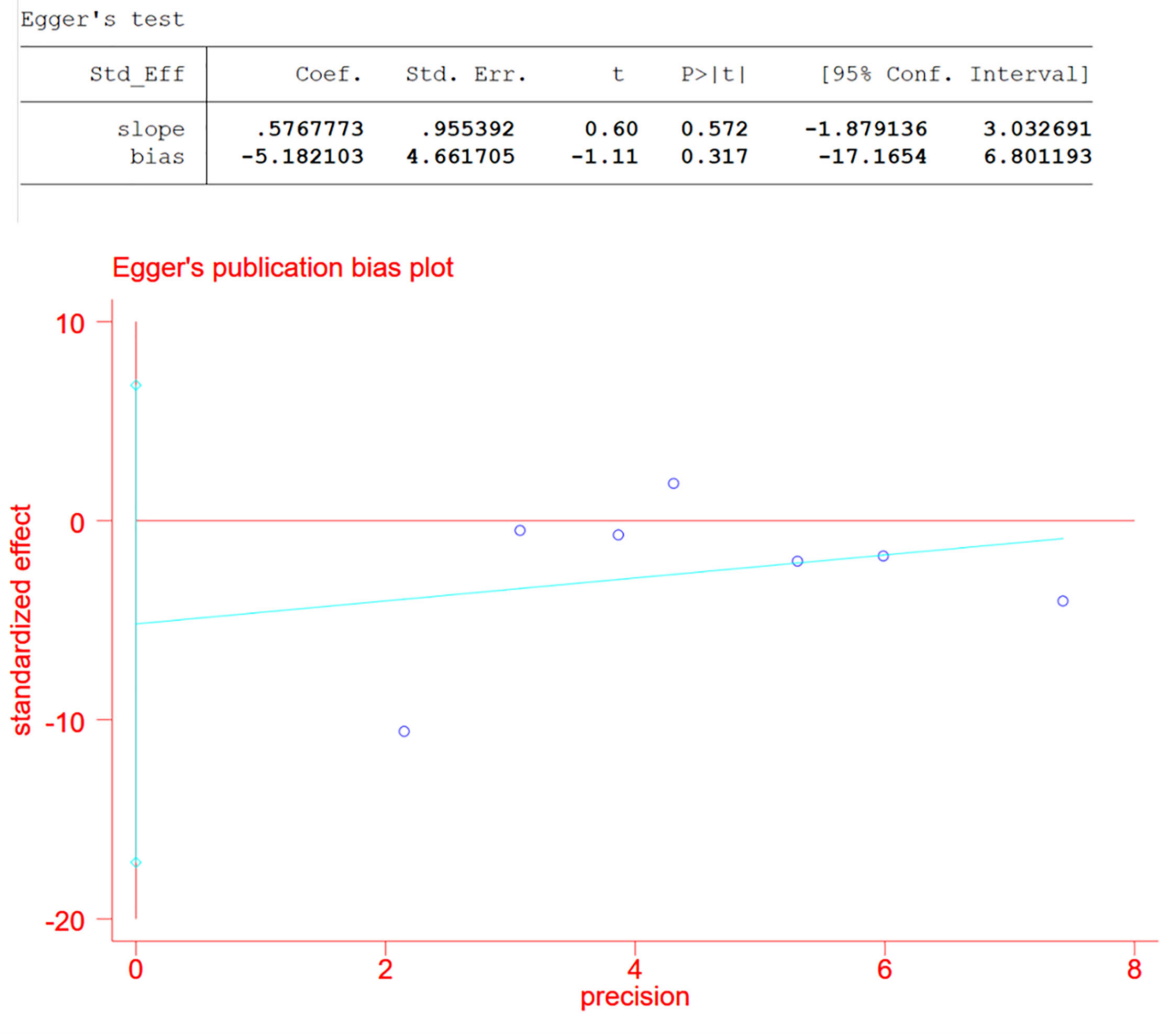
Estimate of publication bias of CMT.

In the pooled analysis of 9 trials, the results showed that combination therapy had a better impact on improving or delaying vision deterioration in BCVA, compared to monotherapy, with a statistically significant between-study heterogeneity (**Figure 3**). Sensitivity analysis was performed in the BCVA group with LogMAR as the unit. Since one of the studies (17) enrolled patients with PDR and DME, while the other studies included patients with or without DME, this might be the reason for the heterogeneity. However, the removal of the study had no effect on the results, which still showed the priority of the combination therapy on improvements of BCVA after the exclusion. The other indicators (NVD, NVE, CMT) (**Figure 4**) all showed a mean overall reduction in favor of PRP plus anti-VEGF treatment, with no significant between-study heterogeneity. For the assessment of publication bias of the CMT group shown in the table (p value >0.05) (**Figure 5**), no significant publication bias was detected.

**Figure 6:**
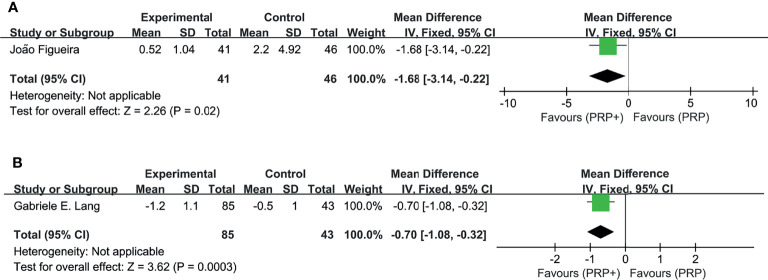
Meta-analyses of PRP+anti-VEGF treatment and PRP monotherapy, comparing changes of NVT (DA) and total retinal volume (mm3). **(A)** NVT (DA). **(B)** Total retinal volume (mm3). Outcomes assessed: BCVA in studies that compared combination treatment with PRP monotherapy. The shaded area is the weight ofthe estimate in proportion to the overall effect.

In addition, two studies separately reported changes of NVT (8) and the mean total retinal volume (10), supporting the findings above (**Figure 6**).

**Figure 7:**
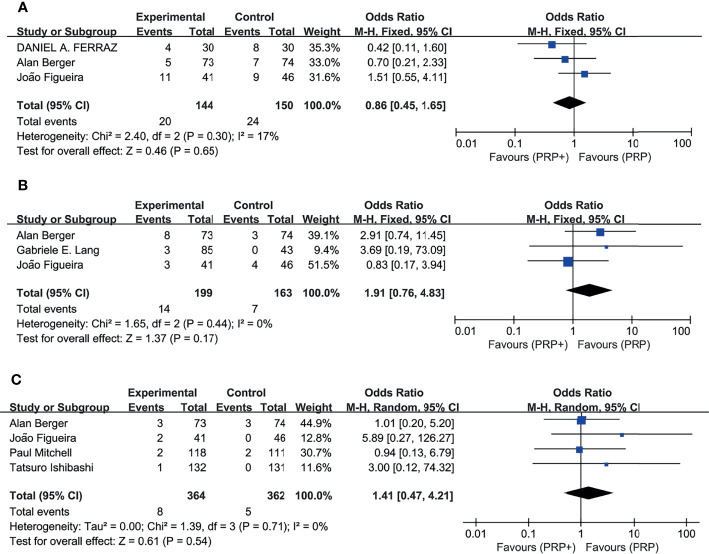
Meta-analyses of PRP+anti-VEGF treatment and PRP monotherapy, comparing incidence of AEs. **(A)** Vitreous hemorrhage. **(B)** Elevation in intraocular pressure. **(C)** Cataract. Outcomes assessed: incidence of vitreous hemorrhage, elevation in intraocular pressure, and cataract in studies that compared combination treatment with PRP monotherapy. The shaded area is the weight of the estimate in proportion to the overall effect.

6 studies reported the incidence of adverse events, including intraocular or extraocular disorders if present. The pooled analysis (**Figure 7**) showed no significant difference in the RR of the study group compared with the control group, with no significant between-study heterogeneity. Combination therapy or monotherapy had little effect on the incidence of adverse events such as vitreous hemorrhage, elevation in intraocular pressure, and cataract.

## Discussion

In the previous study, anti-VEGF monotherapy has been reported to show potential benefits compared to PRP monotherapy. However, anti-VEGF monotherapy also carries a certain degree of risk for PDR. For example, anti-VEGF treatment is expensive, time-consuming, and not cost-effective. In countries and regions with limited resources, this treatment is easily restricted. In addition, it is easy for patients who are treated exclusively with anti-VEGF to cause complications such as tractional retinal detachment and neovascular glaucoma, leading to blindness (18). Our study showed that the combination therapy is even more beneficial. The PRP plus anti-VEGF treatment is better at delaying vision loss associated with DR and also contributing to the reduction of NVD, NVE, and CMT, compared to PRP monotherapy. It is worth mentioning that application of a more complex therapeutic method did not cause more adverse effects than monotherapy. Previous studies have reported the role of anti-VEGF agents as adjuncts to surgical treatment, regressing neovascularization and minimizing bleeding and complications followed by vitrectomy and endolaser for a vitreous hemorrhage (19). Findings in these meta-analyses proved the well-documented efficacy and safety of anti-VEGF.

Retinal detachments have been observed in some PRP groups, which is likely to be associated with anti-VEGF injections in PDR eyes (20), but studies included in this analysis did not have any cases of this probably because eyes were excluded with fibrovascular proliferation with retinal traction.

Most of the studies identified the included patients as DME, PDR, or high-risk PDR (HR-PDR) (7–14, 16, 17). Retinal ischemia has been reported to be present in high-risk PDR in the previous study (21). Preoperative anti-VEGF injections in DR may reduce the bleeding during an operation and decrease the complications (22). Our findings showed that the combination might benefit patients with DME and PDR, by reducing the incidence of complications. In addition, combination therapy consistently improved BCVA and reduced CMT across all the patients with PDR, including patients with DME. Further studies are needed to confirm the beneficial effect of additional anti-VEGF treatment in NPDR patients.

Our results compared anti-VEGF monotherapy with combination therapy in terms of effects on visual improvement, retinal angiogenesis, and the incidence of adverse events, which would give some feasible suggestions on evidence-based clinical practice. A limitation of this analysis is that all the follow-up periods are less than 1 year. Longer follow-up studies may establish the benefit or adverse effects more clearly. Another limitation is the fact that the difference between the DME and non-DME groups cannot be concluded from the data in the RCTs, due to the short follow-up duration or the small sample sizes. The long-term results from relevant studies further showed that response to anti-VEGF is sustained through the period of 2 years (23), and 3 years (24), and the longer the duration, the fewer the injections to control edema, from which we hypothesized that a longer follow-up period would help reveal the relationship between lesion severity and response to treatment.

It has been reported that the intensity of anti-VEGF treatment has a statistically significant effect on BCVA, but there is no confirmed clinical evidence (25). Compared with clinical treatment, the injection intensity in clinical trials is often very high, while in clinical practice, the treatment intensity of many treatment plans is not high in the first year resulting in poorer visual effect and more inaccurate estimation.

In conclusion, Intravitreal anti-VEGF agents for a certain duration are effective as adjunctive treatment to PRP with less foveal thickness, early and greater rate of retinal neovessel regression, and better vision improvement rates than PRP alone in patients of DR.

## Author Contributions

WZ was in charge of the writing of manuscript and analyzing of data while JG was in charge of collecting the data. AS was the corresponding author. All authors contributed to the article and approved the submitted version.

## Conflict of Interest

The authors declare that the research was conducted in the absence of any commercial or financial relationships that could be construed as a potential conflict of interest.

## Publisher’s Note

All claims expressed in this article are solely those of the authors and do not necessarily represent those of their affiliated organizations, or those of the publisher, the editors and the reviewers. Any product that may be evaluated in this article, or claim that may be made by its manufacturer, is not guaranteed or endorsed by the publisher.
